# Index Volume 1 1979

**DOI:** 10.1155/S1463924680000181

**Published:** 1980


### Index Volume 1 1979 – Subject Index (Main articles printed in bold)

A

**Advanced software concepts for employing microcomputers in the laboratory**, B. Scott Tilden and M. Bonner Denton, April 1979, 128.

Analytical Quality Control *(Meeting Report)*, D. Porter, July 1979, 229.

**An automated distillation method for the determination of diacetyl in beer: a comparison of analysis by Auto Analyser and gas chromatography**, J. C. Buitjen and B. Holm, January 1979, 88.

**An automated sampler for high temperature headspace gas chromatography**, J. B. Pausch, R. E. Arner and R. P. Lattimer, October 1978, 22.

**An automatic solution-making device for quality control of dyestuffs: design philosophy and implementation**, Hans-Christian Mez and Gero Michel, July 1979, 189.

**An automatic system for kinetic clinical analysis**, C. Riley, C. F. Darby, D. E. Tutt and D. F. Rocks, January 1979, 77.

**An evaluation of the Kem-O-Mat programmable discrete analyser**, Geoffrey C. Seymour, April 1979, 140.

**An improved automatic liquid injection apparatus for use on a carbon analyser**, R. A. Van Steenderen, January 1979, 91.

**A microcomputer automated recording spectropolarimeter**, Victor C. Zadnik, James L. Scott, Robert Megargle, Julius Kerkay and Karl H. Pearson, July 1979, 206.

**A microprocessor-controlled liquid chromatograph/atomic absorption sampling system**, Thomas M. Vickrey and William Eue, July 1979, 198.

**A simple inert pump for use with concentrated acids in automatic analysis**, *(Short Communication)*, R. G. Lidzey, October 1978, 42.

**Assessment of the Chemetrics analyser**, Robyn White, N. Potezney, T. D. Geary, D. Elston and B. Fuller, October 1978, 40.

**Assessment of the ENI Gemeni microprocessor-controlled centrifugal analyzer**, N. Potezny, R. G. White and T. D. Geary, July 1979, 202.

**A total systems approach to laboratory automation**, Peter B. Stockwell, July 1979, 216.

**Automated determination of hydrogen cyanide, acrolein and total aldehydes in the gas phase of tobacco smoke**, *(Short Communication)*, W. S. Rickert and P. B. Stockwell, April 1979, 152.

**Automated solid-liquid extraction system**, H. Bartels, R. D. Werder, W. Schumann and R. W. Arndt, October 1978, 28.

Automation and Mechanization of Column Operations in Liquid Chromatography, *(Book Review)*, B. Meloun, April 1979, 158.

Automation comes of age *(Commentary)*, P. B. Stockwell, October 1978, 3.

**Automated dissolution rate analysis of iron in some vitamin preparations**, *(Short Communication)*, Bo Karlberg and Sidsel Thelander, April 1979, 149.

Automation in Atomic Spectroscopy, *(Meeting Report)*, D. G. Porter, January 1979, 104.

**Automation in clinical chemistry: developments and recent trends**, F. L. Mitchell, October 1978, 7.

Automation in Clinical Chemistry, *(Meeting Report)*, R. Galimany and F. L. Mitchell, July 1979, 227.

Automation in the measurement of corrosion, *(Meeting Report)*, C. J. Jackson, April 1979, 157.

C

Chromatography Discussion Group, *(Meeting Report)*, W. Lancaster, July 1979, 230.

Chromatography Symposium *(Meeting Report)*, Peter B. Stockwell, October 1978, 43.

**Clinical laboratory evaluation of the Orion SS-20 ionized calcium analyser**, Patrick Ferreira and Alan M. Bold, January 1979, 94.

Compendium of Analytical Nomenclature, *(Book Review)*, Edited by H. M. N. H. Irving, H. Freiser and T. S. West, January 1979, 105.

Computers in Mass Spectrometry, *(Book Review)*, J. R. Chapman, January 1979, 105.

Computing in Clinical Laboratories, *(Book Review)*, Edited by F. Siemaszko, October 1978, 45.

D

**Design principles of a computer controlled multiplexed absorptiometer for reaction rate analysis**, M. Snook, A. Renshaw, J. M. Rideout, D. J. Wright, J. Baker and J. Dickins, January 1979, 72.

**Developments in the philosophy of automatic analysis**, P. B. Stockwell, October 1978, 10.

E

From the Editor's desk, *(Commentary)*, P. B. Stockwell, January 1979, 63.

From the Editor's desk, *(Commentary)*, P. B. Stockwell, April 1979, 119.

From the Editor's desk, *(Commentary)*, Peter B. Stockwell, July 1979, 181.

Education for automation *(Commentary)*, H. V. Malmstadt, April 1979, 119.

Evaluation and optimisation of laboratory methods and analytical procedures, *(Book Review)*, D. L. Massart, A. Dijkstra and L. Kaufman, January 1979, 105.

H

**High-speed, automatic dispenser/diluter or dual pipetter/mixer**, David L. Drottinger, Michael S. McCracken and Howard V. Malmstadt, October 1978, 15.

Laboratory Management and Automation, *(Meeting Report)*, J. Holme, January 1979, 103.

M

Managing Management Information Systems, *(Book Review)*, Phillip Ein Dor and Eli Seger, April 1979, 158.

Microprocessors and the Chemist, *(Meeting Report)*, T. Lenea Goad, January 1979, 104.

P

**Practical and organisational problems in the testing of clinical laboratory instrument**, L. B. Roberts, October 1978, 32.

**Principles of design, supply and usage of clinical laboratory equipment for primary health care in developing countries**, M. Hjelm, S. S. Brown and F. L. Mitchell, July 1979, 214.

Q

Quality Control in the Clinical Laboratory: A Procedural Text *(Book Review)*, Paul J. Ottaviano and Arthur F. Disalvo, October 1978, 45.

R

**Report of a WHO-sponsored trial of MonA and PotLab colorimeters**, *(Short communication)*, J. M. Rideout, July 1979, 223.

S

Safety and Automation, *(Meeting Report)*, D. G. Porter, October 1978, 44.

**Short technical description of the MonA and PotLab colorimeters**, *(Short communication)*, S. N. Pocock and J. M. Rideout, July 1979, 222.

**Specification and production of prototype automatic instrumentation**, J. E. Carlyle, January 1979, 69.

T

**Teaching a graduate level course in automatic chemical analysis**, Richard F. Browner, April 1979, 125.

**The design of a microprocessor system for automatic signal averaging and percentage purity calculations coupled to a nuclear magnetic resonance spectrometer**, F. Morley, I. K. O'Neill, M. A. Pringuer and P. B. Stockwell, January 1979, 83.

**The measurement of splashover and carryover in centrifugal analyzers** Peter Henry, July 1979, 195.

The Trend Towards Devolution in Clinical Chemistry, *(Commentary)*, F. L. Mitchell, July 1979, 179.

**The use of a microcomputer for flexible automation of a liquid chromatograph**, A. D. Mills, I. Mackenzie and R. J. Dolphin, April 1979, 134.

**The Vickers SP 120 analyser: an instrument evaluation**, B. P. Ager, R. Gentle, E. W. Ingarfill and A. L. Tarnoky, October 1978, 36.

Third European Congress of Clinical Chemistry, *(Meeting Report)*, Mark S. Stoll, July 1979, 226.

Thirtieth Pittsburgh Conference, *(Meeting Report)*, Peter B. Stockwell, April 1979, 155.

Topics in Automatic Chemical Analysis, *(Book Review)*, Edited by J. K. Foreman and P. B. Stockwell, July 1979, 231.

W

Why automation lags, *(Commentary)*, R. W. Arndt, January 1979, 63.

### Index Volume 1 1979

### – Author Index

A

**Ager, B. P., Gentle, R., Ingarfill, E. W. and Tarnoky, A. L.** The Vickers SP 120 analyser: an instrument evaluation, October 1978, 36.
**Arndt, R. W.** Why automation lags *(Commentary)*, January 1979, 63.
**Arndt, R. W.** *see* Bartels, H.
**Arner, R. E.** *see* Pausch, J. B.

B

**Baker, J.** *see* Snook, M.
**Bartels, H., Werder, R. D., Schumann, W. and Arndt, R. W.** Automated solid-liquid extraction system, October 1978, 28.
**Bold, Alan M.** *see* Ferreira, Patrick.
**Brown, S. S.** *see* Hjelm, M.
**Browner, Richard F.** Teaching a graduate level course in automatic chemical analysis, April 1979, 125.
**Buitjen, J. C. and Holm, B.** An automated distillation method for the determination of diacetyl in beer: a comparison of analysis by Auto Analyser and gas chromatography, January 1979, 88.

C

**Carlyle, J. E.** Specification and production of prototype automatic instrumentation, January 1979, 69.
**Chapman, J. R.** Computers in Mass Spectrometry *(Book Review)*, January 1979, 105.

D

**Darby, C. F.** *see* Riley, C.
**Denton, M. Bonner** *see* Tilden, Scott B.
**Dickins, J.** *see* Snook, M.
**Dijkstra, A.** *see* Massart, D. L.
**Disalvo, Arthur F.** *see* Ottaviano, Paul J.
**Dolphin, R. J.** *see* Mills, A. D.

E

**Ein, Dor Phillip and Seger, Eli.** Managing Management Information Systems *(Book Review)*, April 1979, 158.
**Elston, D.** *see* White, Robyn.
**Eue, William** *see* Vickrey, Thomas M.

F

**Ferreira, Patrick and Bold, Alan M.** Clinical laboratory evaluation of the Orion SS-20 ionized calcium analyser, January 1979, 94.
Edited by **Foreman, J. K. and Stockwell, P. B.** Topics in Automatic Chemical Analysis *(Book Review)*, July 1979, 231.
**Freiser, H.** *see* Irving, H. M. N. H.
**Fuller, B.** *see* White, Robyn.

G

**Galimany, R. and Mitchell, F. L.** Automation in Clinical Chemistry *(Meeting Report)*, July 1979, 227.
**Geary, T. D.** *see* Potezny, N.
**Geary, T. D.** *see* White, Robyn.
**Gentle, R.** *see Ager, B. P.*
**Goad, T. Leanea** Microprocessors and the Chemist *(Meeting Report)*, January 1979, 104.

H

**Henry, Peter.** The measurement of splashover and carryover in centrifugal analyzers, July 1979, 195.
**Hjelm, M., Brown, S. S. and Mitchell, F. L.** Principles of design, supply and usage of clinical laboratory equipment for primary health care in developing countries, July 1979, 214.
**Holm, B.** *see* Buitjen, J. C.
**Holme, J.** Laboratory Management and Automation *(Meeting Report), January 1979, 103.*

I

**Ingarfill, E. W.** *see* Ager, B. P.
**Irving, H. M. N. H., Freiser, H. and West, T. S.** Compendium of Analytical Nomenclature *(Book Review)*, January 1979, 105.

J

**Jackson, C. J.** Automation in the measurement of corrosion *(Meeting Report)*, April 1979, 157.

K

**Karlberg Bo and Thelander Sidsel.** Automated dissolution rate analysis of iron in some vitamin preparations *(Short Communication)*, April 1979, 149.
**Kaufman, L.** *see* Massart, D. L.
**Kerkay Julius** *see* Zadnik Victor C.
**Krottinger David L., McCracken Michael S., Malmstadt Howard, V.** High-speed, automatic dispenser/diluter or dual pipetter/mixer, October 1978, 15.

L

**Lancaster, W.** Chromatography Discussion Group *(Meeting Report)*, July 1979, 230.
**Lattimer, R. P.** *see* Pausch, J. B.
**Lidzey, R. G.** A simple inert pump for use with concentrated acids in automatic analysis *(Short Communication)*, October 1978, 42.

M

**Mackenzie, I.** *see* Mills, A. D.
**Malmstadt Howard, V.** *see* Krottinger David, L.
**Malmstadt, H. V.** Education for automation *(Commentary)*, April 1979, 119.
**Massart, D. L., Dijkstra, A. and Kaufman, L.** Evaluation and optimisation of laboratory methods and analytical procedures *(Book Review)*, January 1979, 105.
**McCracken Michael, S.** *see* Krottinger, David L.
**Meloun, B.** Automation and Mechanization of Column Operations in Liquid Chromatography *(Book Review)*, April 1979, 158.
**Megargle, Robert** *see* Zadnik, Victor C.
**Mez, Hans-Christian and Michel, Gero.** An automatic solution-making device for quality control of dyestuffs: design philosophy and implementation, July 1979, 189.
**Michel, Gero** *see* Mez, Hans-Christian.
**Mills, A. D., Mackenzie, I. and Dolphin, R. J.** The use of a microcomputer for flexible automation of a liquid chromatograph, April 1979, 134.
**Mitchell, F. L.** Automation in clinical chemistry: developments and recent trends, October 1978, 7.
**Mitchell, F. L.** The Trend Towards Devolution in Clinical Chemistry *(Commentary)*, July 1979, 179.
**Mitchell, F. L.** *see* Galimany, R.
**Mitchell, F. L.** *see* Hjelm, M.
**Morley, F., O'Neill, I. K., Pringuer, M. A., Stockwell, P. B.** The design of a microprocessor system for automatic signal averaging and percentage purity calculations coupled to a nuclear magnetic resonance spectrometer, January 1979, 83.

O

**O'Neill, I. K.** *see* Morley, F.
**Ottaviano, Paul J. and Disalvo, Arthur F.** Quality Control in the Clinical Laboratory: A Procedural Text *(Book Review)*, October 1978, 45.

P

**Pausch, J. B., Arner, R. E. and Lattimer, R. P.** An automated sampler for high temperature headspace gas chromatography, October 1978, 22.
**Pearson, Karl H.** *see* Zadnik, Victor C.
**Pocock, S. N. and Rideout, J. M.** Short technical description of the MonA and PotLab colorimeters *(Short communication)*, July 1979, 222.
**Porter, D.** Analytical Quality Control *(Meeting Report)*, July 1979, 229.
**Porter, D. G.** Automation in Atomic Spectroscopy *(Meeting Report)*, January 1979, 104.
**Porter, D. G.** Safety and Automation *(Meeting Report)*, October 1978, 44.
**Potezny, N., White, R. G. and Geary, T. D.** Assessment of the ENI Gemeni microprocessor-controlled centrifugal analyzer, July 1979, 202.
**Potezney, N.** *see* White, Robyn.
**Pringuer, M. A.** *see* Morley, F.

R

**Renshaw, A.** *see* Snook, M.
**Rickert, W. S. and Stockwell, P. B.** Automated determination of hydrogen cyanide, acrolein and total aldehydes in the gas phase of tobacco smoke *(Short Communication)*, April 1979, 152.

**Rideout, J. M.** Report of a WHO-sponsored trial of MonA and PotLab colorimeters *(Short communication)*, July 1979, 223.
**Rideout, J. M.** *see* Pocock, S. N.
**Rideout, J. M.** *see* Snook, M.
**Riley, C., Darby, C. F., Tutt, D. E. and Rocks, D. F.** An automatic system for kinetic clinical analysis, January 1979, 77.
**Rocks, D. F.** *see* Riley, C.
**Roberts, L. B.** Practical and organisational problems in the testing of clinical laboratory instrument, October 1978, 32.

S
**Schumann, W.** *see* Bartels, H.
**Scott, James L.** *see* Zadnik, Victor C.
**Seger, Eli** *see* Ein, Dor Phillip.
**Seymour, Geoffrey C.** An evaluation of the Kem-0-Mat programmable discrete analyser, April 1979, 140.
Edited by **Siemaszko, F.** Computing in Clinical Laboratories *(Book Review)*, October 1978, 45.
**Snook, M., Renshaw, A., Rideout, J. M., Wright, D. J., Baker, J. and Dickins, J.** Design principles of a computer controlled multiplexed absorptiometer for reaction rate analysis, January 1979, 72.
**Stockwell, Peter B.** A total systems approach to laboratory automation, July 1979, 216.
**Stockwell, P. B.** Automation comes of age *(Commentary)*, October 1978, 3.
**Stockwell, Peter B.** Chromatography Symposium *(Meeting Report)*, October 1978, 43.
**Stockwell, P. B.** Developments in the philosophy of automatic analysis, October 1978, 10.
**Stockwell, P. B.** From the Editor's desk *(Commentary)*, January 1979, 63.
**Stockwell, P. B.** From the Editor's desk *(Commentary)*, April 1979, 119.
**Stockwell, Peter B.** From the Editors Desk *(Commentary)*, July 1979, 181.
**Stockwell, Peter B.** Thirtieth Pittsburgh Conference *(Meeting Report)*, April 1979, 155.
**Stockwell, P. B.** *see* Foreman, J. K.
**Stockwell, P. B.** *see* Morley, F.
**Stoll, Mark S.** Third European Congress of Clinical Chemistry *(Meeting Report)*, July 1979, 226.

T
**Tarnoky, A. L.** *see* Ager, B. P.
**Thelander, Sidsel** *see* Karlberg, Bo.
**Tilden, Scott B. and Denton, M Bonner.** Advanced software concepts for employing micricomputers in the laboratory, April 1979, 128.
**Tutt, D. E.** *see* Riley, C.

V
**Van, Steenderen, R. A.** An improved automatic liquid injection apparatus for use on a carbon analyser, January 1979, 91.
**Vickrey, Thomas M. and Eue, William.** A microprocessor-controlled liquid chromatograph/atomic absorption sampling system, July 1979, 198.

W
**Werder, R. D.** *see* Bartels, H.
**West, T. S.** *see* Irving, H. M. N. H.
**White, Robyn, Potezney, N., Geary, T. D., Elston, D. and Fuller B.** Assessment of the Chemetrics analyser, October 1978, 40.
**White, R. G.** *see* Potezny, N.
**Wright, D. J.** *see* Snook, M.

Z
**Zadnik, Victor C., Scott, James L., Megargle, Robert, Kerkay, Julius and Pearson, Karl H.** A microcomputer automated recording spectropolarimeter, July 1979, 206.

### Notes for Contributors

### Presentation of manuscripts

Manuscripts should be typed (double-spaced) on one side of the paper only and with generous margins. The title should be brief and informative avoiding the word "new" and its synonyms. The full list of authors with their affiliations and full address(es) should appear on the title page. On a separate sheet an abstract of no more than 150 words is required. This should succinctly describe the scope of the contribution and highlight significant findings or innovations. It should be written in a style which can easily be translated into French and German.

*The Concise Oxford Dictionary* and Fowler's *Modern English Usage* (both published by Oxford University Press) should be used as the standard for spelling and grammar. Abbreviations should be limited to those generally recognised, or where a frequently occuring term is abbreviated it should, in the first instance, be explained thus "flow injection analysis (FIA) ..." and the abbreviation used thereafter. Abbreviations, for standard measures and units should follow SI recommendations. There are various publications giving guidance on the use of SI units.

References should be indicated in the text by numerals following the author's name, i.e. Skeggs [6]. On a separate sheet of paper, list all references in numerical order thus:

[6] Skeggs, L.T., *American Journal of Clinical Pathology*, 1959, **28**, 311.

Note that journal titles are given in full. Where there is more than one author, the form Foreman et al. should be used in the text but all authors should be named in the list of references. When reference is made to a chapter in a book the reference should take the following form:

[7] Malmstadt, H.V. in "Topics in Automatic Chemistry" Ed. Stockwell P.B. and Foreman J.K. 1978 Horwood, Chichester, pp. 68-70.

Only work which has been published or has been accepted for publication should be cited. Avoid the citation of documents which are subject to restricted circulation, patent literature, unpublished work and personal communications. The latter can be mentioned in the text in parenthesis.

To illustrate a paper line diagrams are preferred to photographs. Photographs should only be used when they significantly add to the discussion. Diagrams, charts and graphs should be carefully drawn in black ink on stout card or heavy quality tracing paper. Most illustrations are reduced for publication; to allow for this originals should be between 16 and 36 cm wide (the depth must not exceed 50 cm). The lettering of diagrams should be sufficiently clear to withstand reduction. Except in the case of proper names, all lettering should be in lower case print. If photographs are used they must be supplied in the form of clear, unmounted, glossy, black and white prints. "Instant" photographs are not normally acceptable. All illustrations must be identified on the reverse showing the figure number and the author's name.

Each illustration should have a fully explanitory caption. Captions should be typed together on a separate sheet of paper; they must *not* be an inseparable part of the illustration.

